# An update on gastrointestinal stromal tumors (GISTs) with a focus on extragastrointestinal stromal tumors (EGISTs)

**DOI:** 10.1093/gastro/goaf068

**Published:** 2025-07-23

**Authors:** Fatima Usama, Rohullah Rasikh, Khawaja Hassam, Mansoor Rahman, FNU Khalil Ur Rehman, Iman Waheed Khan, Daryl T -Y Lau

**Affiliations:** Internal Medicine Department, Yale Waterbury, Waterbury, CT, USA; Liver Center, Department of Medicine, Beth Israel Deaconess Medical Center, Harvard Medical School, Boston, MA, USA; Internal Medicine Department, Lahey Hospital and Medical Center, Burlington, MA, USA; Internal Medicine Department, Hamilton Medical Center, Dalton, GA, USA; Liver Center, Department of Medicine, Beth Israel Deaconess Medical Center, Harvard Medical School, Boston, MA, USA; Liver Center, Department of Medicine, Beth Israel Deaconess Medical Center, Harvard Medical School, Boston, MA, USA; Internal Medicine Department, Reading Hospital, Tower Health, Reading, PA, USA; Liver Center, Department of Medicine, Beth Israel Deaconess Medical Center, Harvard Medical School, Boston, MA, USA

**Keywords:** gastrointestinal stromal tumor, extragastrointestinal stromal tumor, mesenchymal gastroenterological tumors, KIT mutation, PDGFRA mutation

## Abstract

Gastrointestinal stromal tumors (GISTs) originate from mesenchymal cells and account for ∼1% of primary malignant tumors in the digestive system. They are diagnosed based on characteristic immunohistochemical staining pattern, including CD117 and DOG1, as well as genetic analysis for mutations in the KIT and platelet-derived growth factor receptor α genes. Extragastrointestinal stromal tumors (EGISTs) share very similar morphology with GISTs but arise outside the gastrointestinal tract. The most common locations for EGISTs are the omentum, mesentery, retroperitoneum, and pancreas, followed by the liver, vagina, and prostate. The mean age of presentation of these tumors is in the sixth decade of life and tumor dimensions at different locations typically range from 7 to 15.8 cm. Most of these tumors are unifocal and of the spindle cell type. GISTs generally have a better prognosis than EGISTs, with cumulative 5-year survival rates of 85% for GISTs and 38%–60.9% for EGISTs. Among EGISTs, omental tumors have higher overall survival than mesenteric or retroperitoneal tumors. Additionally, age of >60 years, male sex, larger tumor size, higher mitotic rate, and nuclear pleomorphism are associated with worse prognosis in EGISTs.

## Introduction

Gastrointestinal stromal tumors (GISTs) are the most common non-epithelial tumors of the gastrointestinal tract. These tumors, originating from mesenchymal cells, account for ∼1% of all primary malignant tumors in the digestive system [1]. Initially, GISTs were classified as smooth-muscle tumors [2]. However, Mazur and Clark [3] discovered that the tumors in the stomach that were previously labeled as leiomyomas did not display the immunohistochemical features of Schwann cells (S100 protein-negative) and lacked the ultrastructural characteristics of smooth-muscle cells. They therefore introduced the term “gastric stromal tumors” as a neutral designation that could be applied to similar tumors in the intestines. Subsequently, the term “GIST” has become widely used to describe these specific mesenchymal tumors of the gastrointestinal tract [3]. It is believed that GISTs arise from the interstitial cells of Cajal (ICC), which mediate peristalsis in the wall of the gastrointestinal tract [4].

GISTs occur predominantly in middle-aged and older individuals, with a peak incidence in the seventh decade of life. The prevalence is similar in both genders [5]. These tumors can arise anywhere along the gastrointestinal (GI) tract, with the most common location in the stomach (51%), followed by the small intestine (36%), colon (7%), rectum (5%), and, least frequently, the esophagus (1%) [6].

A rare subgroup of GISTs, known as the extragastrointestinal stromal tumors (EGISTs), arises outside the GI tract. An EGIST is identified by using the standard pathological criteria including immunohistochemistry and molecular gene analysis consistent with GIST [7]. Some studies suggest that EGISTs may represent distant or implantation metastases of GISTs, while others propose that they constitute a distinct subtype within the GIST spectrum [8, 9]. Epidemiological data on the incidence of EGISTs are incomplete, but they are estimated to account for ∼10% of all GISTs [10].

In this review, we performed a systematic literature analysis of these lesser-known EGISTs and provide a comprehensive update on the key features of GISTs.

## Epidemiology and clinical presentation

GISTs are the most prevalent type of mesenchymal tumors, accounting for ∼80% of GI mesenchymal tumors, 5%–6% of sarcomas, 14% of all malignancies in the small intestine, and 0.1% of all malignancies in the colon [11–13]. According to a systematic review of 29 studies conducted across 19 countries, the annual incidence rate of GISTs ranged from 10 to 15 cases per 1 million people [5]. The highest incidence rates were recorded in China, Korea, and Norway, with 19–22 cases per million per year, while the lowest rates were in the Czech Republic and Slovakia, with 5.2 cases per million per year [5]. In the USA and Canada, the incidence rates are approximately seven to eight cases per million per year [5, 14].

Based on a systematic review of 15 studies on GISTs, including a total of 1,997 cases, the majority (81.3%) of patients presented with symptomatic disease, while 18.7% were asymptomatic and diagnosed incidentally [5]. The most common presenting complaint was overt or occult gastrointestinal bleeding. Other reported symptoms included abdominal pain or discomfort, early satiety, bloating, obstructive jaundice, dysphagia, fever, and anemia-related presentations such as fatigue and palpitations [15].

## Genetic alterations in GIST

Initially, GISTs were thought to be smooth-muscle tumors based on their histologic characteristics. The identification of the proto-oncogene c-*KIT*, the gene encoding the receptor tyrosine kinase protein known as tyrosine-protein kinase KIT/CD117 (cluster of differentiation 117), and the discovery of the origin of GISTs from ICCs led to the recognition of GISTs as a distinct entity [16].

GISTs are classified into two groups based on their mutation profiles (Table 1) [17, 18]. The first group consists of KIT/PDGFRA (platelet-derived growth factor receptor α) mutant types. The second group encompasses KIT/PDGFRA-negative tumors, referred to as wild-type GISTs, which are further subdivided into succinate dehydrogenase (SDH)-deficient and SDH-competent according to SDH complex subunit B (SDHB) immunohistochemical (IHC) expression. Most of the mutations reported in GISTs are sporadic, although a minority involve germ-line mutations related to specific syndromes or familial GISTs.

**Table 1.** Molecular classification of GISTs [17, 18].

Genetic type	Frequency (%)	Anatomic distribution
**KIT mutation**	80–85	
Exon 11 Exon 9 Exons 13, 14, 17	70	All sites
	12–15	Small intestine
	Rare	All sites
**PDGFRA mutations**	5–7	
Exon 18 Exons 14, 12	62.6	Stomach, mesentery
	Rare	
**Wild-type**	5–10	
SDH-deficient	7.5	Stomach
SDH-competent NF1-related BRAF-related	2.5	All sites

Around 80%–92% of patients diagnosed with GISTs have mutations in either the KIT or the PDGFRA genes [18]. The *KIT* gene in humans is located on chromosome 4q12-13. Its products are part of the receptor tyrosine kinase class III family, which also includes PDGFRA and PDGFRB (platelet-derived growth factor receptor beta) [19]. This class III subclass has distinct features; the extracellular region contains five immunoglobulin (Ig)-like domains, in addition to a transmembrane domain and a tyrosine kinase domain located inside the cell [20]. The tyrosine kinase domain is divided into two parts by an insert region. Recent studies have shown that the first three Ig-like domains of KIT, located at the N-terminal end, are responsible for binding with ligands [21]. The ligand binding to the extracellular domain of the KIT receptor activates downstream signaling pathways that promote cell survival, growth, and proliferation [22].

Mutations in the KIT gene in GISTs are identified in different gene exons and include point mutations, deletions, or insertions, all of which can occur in sporadic and hereditary cases. There is no specific hotspot for KIT mutations in GISTs, but some exons are more commonly affected, as shown in Table 1. The majority (∼70%) of the KIT mutations in GISTs are in exon 11, which encodes the juxtamembrane domain of the receptor. This region usually has an autoinhibitory function on kinase activation, which is alleviated by the mutation. Mutations in exon 9, affecting the extracellular ligand-binding domain, are detected in 12%–15% of cases. Primary mutations in the kinase domain, such as in exon 13 (ATP-binding) and exon 17 (activation loop), are rare [22]. Similarly to the KIT mutations, there is no single hotspot for PDGFRA mutations in GISTs. These mutations can be identified in exons 12, 14, and 18. In a study of 289 GISTs with PDGFRA mutations, the exon 18 D842V mutation was the most frequent, noted in 62.6% of cases [23]. GISTs with PDGFRA mutations exhibit epithelioid morphology and arise exclusively in the stomach. In contrast, most GISTs with spindle cells have KIT mutations and can arise at any site in the GI tract [18].

Approximately 5%–10% of GISTs lack mutations in either the KIT or PDGFRA genes. They are collectively grouped as *KIT/PDGFRA*-wild-type (WT) GISTs. In a comprehensive study [24] examining the molecular subtypes of *KIT/PDGFRA*-WT GISTs, it was found that the majority of those cases had alterations in SDH, also known as mitochondrial complex II, that resulted in the loss of SDHB expression. The study defined three distinct molecular subtypes in 84 cases: SDHX mutations (66%), SDHC promoter hypermethylation (22%), and SDH competence (12%) [24]. The former two subtypes are associated with SDH deficiency and present predominantly in the stomach. The SDH-competent subtype can be further divided into Neurofibromatosis type 1 (NF1) mutations and BRAF V600E mutations, which can arise in various parts of the gastrointestinal tract.

### Genetic similarities between GISTs and EGISTs

GISTs and EGISTs share key molecular features, most notably mutations in the KIT and PDGFRA genes, which lead to constitutive activation of receptor tyrosine kinases and drive tumorigenesis [18, 25]. Immunohistochemically, both tumor types typically express CD117 (KIT protein) and DOG1 [26, 27], and both harbor KIT mutations in exon 11 [22, 28]. However, EGISTs exhibit some distinctive patterns. For example, one study reported that PDGFRA mutations appeared more frequently in omental EGISTs (up to ∼52%) compared with typical GISTs (∼8%–10%) [29]. Approximately 10%–15% of GISTs are wild-type for both KIT and PDGFRA genes, and may harbor alternative mutations in genes such as SDH, NF1, or BRAF [24]. However, EGISTs carry a poorer prognosis and are less well studied and characterized at the molecular level, underscoring the importance of more site-specific genetic profiling.

## Pathology of GISTs

The diagnosis of GISTs is established through histopathology, IHC, and identifying disease-specific mutations unique to these neoplasms.

### Histology

Histologically, GISTs are classified into three types: spindle cell (70%), epithelioid (20%), and mixed (10%). While histology raises suspicion, definitive diagnosis requires immunohistochemical and molecular confirmation [30]. Spindle cell GISTs are composed of eosinophilic cells with slightly paler cytoplasm than leiomyomas [31]. Epithelioid GISTs consist of rounded epithelioid cells with clear, eosinophilic cytoplasm. These tumors are predominantly found in the stomach and more often lack KIT expression, frequently harboring PDGFRA mutations [32]. Mixed-type GISTs contain both types of cells, fusiform (spindle) and epithelioid.

### Immunohistochemistry

Immunohistochemically, CD34 was previously considered the best marker for GISTs [33]; however, the major diagnostic markers now are CD117 (KIT) and Discovered on GIST-1 (DOG1), both of which show high sensitivity, specificity, and diagnostic accuracy (>98%) [15] for GISTs. These markers reliably distinguish GISTs from other sub-epithelial tumors such as leiomyomas, which are negative for CD117 [26, 34, 35]. A protein highly expressed in GIST, called PKC-theta, is undetectable in other mesenchymal or epithelial tumors, including non-GIST KIT-positive tumors [36]. Additional markers exhibited in GIST such as CD34 (60%–70%), PKC-theta (90%), caldesmon (60%–80%), smooth muscle actin (SMA) (30%–40%), S-100 (5%), and desmin (1%–2%) can support diagnosis, but their expression varies by tumor location and they lack the specificity of CD117 or DOG1. Notably, colorectal and esophageal GISTs tend to show consistent CD34 positivity, whereas small bowel tumors more frequently express SMA [37, 38].

**Figure 1.**
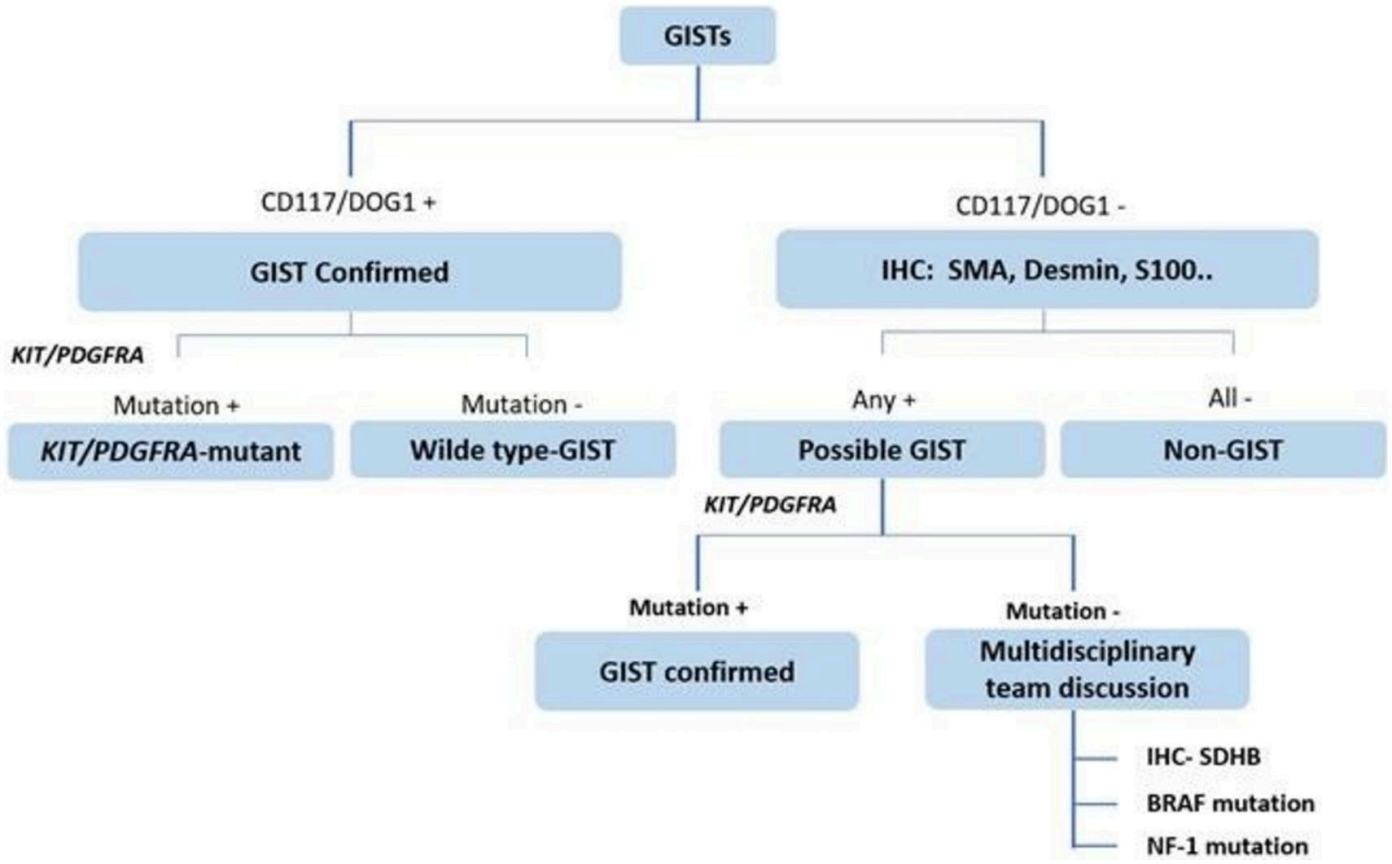
IHC steps involved in confirming the diagnosis of GISTs.

If there is a suspicion of mesenchymal tumors, stepwise IHC should be conducted, as illustrated in Figure 1. In practice, a positive CD117 or DOG1 result confirms GISTs, followed by gene sequencing for KIT and PDGFRA mutations. If both markers are negative, then second-line markers (CD34, SMA, S100, desmin, vimentin, caldesmon) are assessed and gene sequencing is considered if any of these markers is positive. Gene sequencing is used to detect KIT and PDGFRA mutations. Conversely, if all these markers yield negative results, then GIST can be confidently ruled out [18].

## Prognosis of GISTs and EGISTs

The cumulative 5-year survival rate for GISTs is ∼85% [7, 39]. In contrast, the 5-year survival rates for EGISTs are significantly lower, ranging between 38% and 60.9% [7, 40, 41]. The location of EGISTs is an important factor that influences survival. Because EGISTs are situated outside the gastrointestinal tract, they often lack symptoms such as bleeding. As a result, EGISTs are often diagnosed at a later stage, when tumors are larger, the mitotic index is higher, and distant metastases are already present [9, 42].

The primary treatment for localized GISTs is radical resection with negative margins; however, there is a risk of recurrence after surgery [43]. Identification of risk factors for recurrence can help characterize patients who may benefit from adjuvant therapy in addition to surgery and to establish prognosis [43]. There is no separate standardized treatment approach for EGISTs due to their low prevalence. Treatment modalities for EGISTs are generally similar to those for GISTs.

Imatinib (STI-571)—a tyrosine kinase inhibitor known to inhibit the activities of KIT and PDGFRA—is currently the treatment of choice for metastatic and unresectable GISTs. More recently, imatinib has also been recommended as an adjuvant therapy following complete surgical resection of high-risk GISTs to prevent tumor recurrence [44, 45]. Imatinib treatment is typically recommended for 1 year; however, a recent randomized trial demonstrated that extending therapy to 3 years leads to a more favorable recurrence-free survival. These treatment guidelines are also applied to patients with EGISTs [46].

Liu *et al.* [47] utilized the SEER (Surveillance, Epidemiology, and End Results) database between 2000 and 2019 to conduct a detailed survival analysis of GISTs and EGISTs, including a total of 12,627 GIST and 703 EGIST cases. The mean 1-year, 3-year, 5-year, and 10-year overall survival (OS) rates for EGIST patients were 78.3%, 61.9%, 50.5%, and 32.5%, respectively, whereas the corresponding OS rates for GIST patients were 90.6%, 79.8%, 70.3%, and 51.5%, respectively. EGIST patients had significantly poorer OS (hazrad ratio [HR] 1.7, 95% confidence interval [CI]: 1.5–2.0) and cancer-specific survival (CSS) (HR 2.2, 95% CI: 1.8–2.6) than GIST patients (*P *< 0.001) [47]. In a multivariate Cox regression analysis, age, gender, tumor grade, and surgery were identified as independent risk factors for both OS and CSS among EGIST patients. Patients aged >60 years had significantly poorer OS (HR 2.0, 95% CI: 1.6–2.4, *P* < 0.001) and CSS (HR 1.4, 95% CI: 1.1–1.8, *P* = 0.002) compared with younger patients. Similarly, male gender was associated with significantly worse OS (HR 1.3, 95% CI: 1.1–1.6, *P* = 0.007) and CSS (HR 1.3, 95% CI: 1.0–1.7, *P* = 0.020) compared with females. In addition, as shown in a propensity score analysis of EGISTs matched by age, race, sex, year of diagnosis, grade, and tumor size, patients who underwent surgery had significantly higher 5-year OS rates (49.0% vs 39.9%, *P *= 0.035) and CSS rates (63.9% vs 53.0%, *P *= 0.028) than those who did not undergo surgery. Notably, this study did not observe an improvement in OS or CSS among EGIST patients receiving chemotherapy [47].

A meta-analysis including 1,487 GIST patients found that the KIT mutations compared with PDGFRA mutations and wild-type tumors were associated with an increased risk of tumor size of >5 cm and higher mitotic activity. The KIT mutation-positive subgroup also had a significantly higher rate of tumor recurrence (relative risk [RR]= 2.06, 95% CI: 1.37–3.11; *P *= 0.0005) and metastasis (RR = 2.77, 95% CI: 1.64–4.67; *P *= 0.0001) than the KIT mutation-negative subgroup [48].

Reith *et al.* [49] studied the histological parameters of 31 patients with EGISTs. In the multivariable analysis, cellularity (relative risk 2.82), mitotic activity (relative risk 7.46), and necrosis (relative risk 3.75) were independent predictors for adverse outcomes, although statistical significance was not achieved at the 0.05 level 49]. In a study on 28 patients with EGISTs, Kim *et al.* [50] reported that the presence of tumor necrosis, obvious nuclear atypia, and epithelioid or mixed cell type was correlated with worse OS (*P *< 0.05). Yamamoto *et al.* [51] performed a study on 33 patients with EGISTs and found that a higher mitotic rate (>5/50 high-power fields [HPFs]) was significantly associated with both shorter disease-specific survival (*P *= 0.015) and shorter disease-free survival (*P *= 0.014). A summary of the studies has been provided in Table 2 [28–51].

**Table 2.** Overview of studies examining prognostic outcomes.

Study	Number of cases	Main findings with key statistical results	Significance
Liu *et al.* [47]	12,627 GIST 703 EGIST	EGISTs have significantly poorer OS (HR 1.7) and CSS (HR 2.2) vs GIST (*P* < 0.001). Age >60 years, male sex, and lack of surgery were key risk factors. Surgery improved 5-year OS (49% vs 39.9%) and CSS (63.9% vs 53%)	Confirms poorer prognosis of EGIST vs GIST and identifies key clinical predictors, emphasizing surgical benefit Chemotherapy showed no survival benefit
Zong *et al.* [48]	1,487 GIST	KIT mutations (vs PDGFRA mutations and wild-type) associated with increased risk for tumor size >5 cm, higher mitotic activity, recurrence, and metastasis. KIT mutations: recurrence RR = 2.06 (95% CI: 1.37–3.11; *P *= 0.0005); metastasis RR = 2.77 (95% CI: 1.64–4.67; *P *= 0.0001)	Highlights the prognostic impact of KIT mutations, emphasizing the need for mutation-specific risk assessment and closer follow-up in KIT-positive patients
Reith *et al.* [49]	31 EGIST	Cellularity (RR 2.82), mitotic activity (RR 7.46), and necrosis (RR 3.75) were adverse predictors, but not statistically significant at *P *< 0.05	Identifies key pathological predictors of poor outcome; limited by small sample size
Kim *et al.* [50]	28 EGIST	Tumor necrosis, nuclear atypia, epithelioid/mixed histology correlated with worse OS (*P *< 0.05)	Correlates specific histological features with worse survival, reinforcing importance of detailed pathology review
Yamamoto *et al.* [51]	33 EGIST	High mitotic rate (>5/50 HPFs) was significantly associated with shorter disease-specific survival (*P *= 0.015) and disease-free survival (*P *= 0.014)	Confirms high mitotic rate as a robust prognostic marker for survival outcomes
Zhou *et al.* [40]	22 EGIST (omentum, mesentery, retroperitoneum)	Omental EGISTs had the best prognosis; retroperitoneal EGISTs had the worst	Demonstrates variation in prognosis by tumor site, advocating for location-based prognostic models
Lin *et al.* [28]	6 retroperitoneal EGIST	KIT Exon 10 mutation associated with poor prognosis; exon 11 mutation linked with better prognosis due to favorable imatinib response	Links specific genetic mutations to prognosis and treatment response, suggesting value of molecular profiling

## Extragastrointestinal stromal tumors

**Figure 2.**
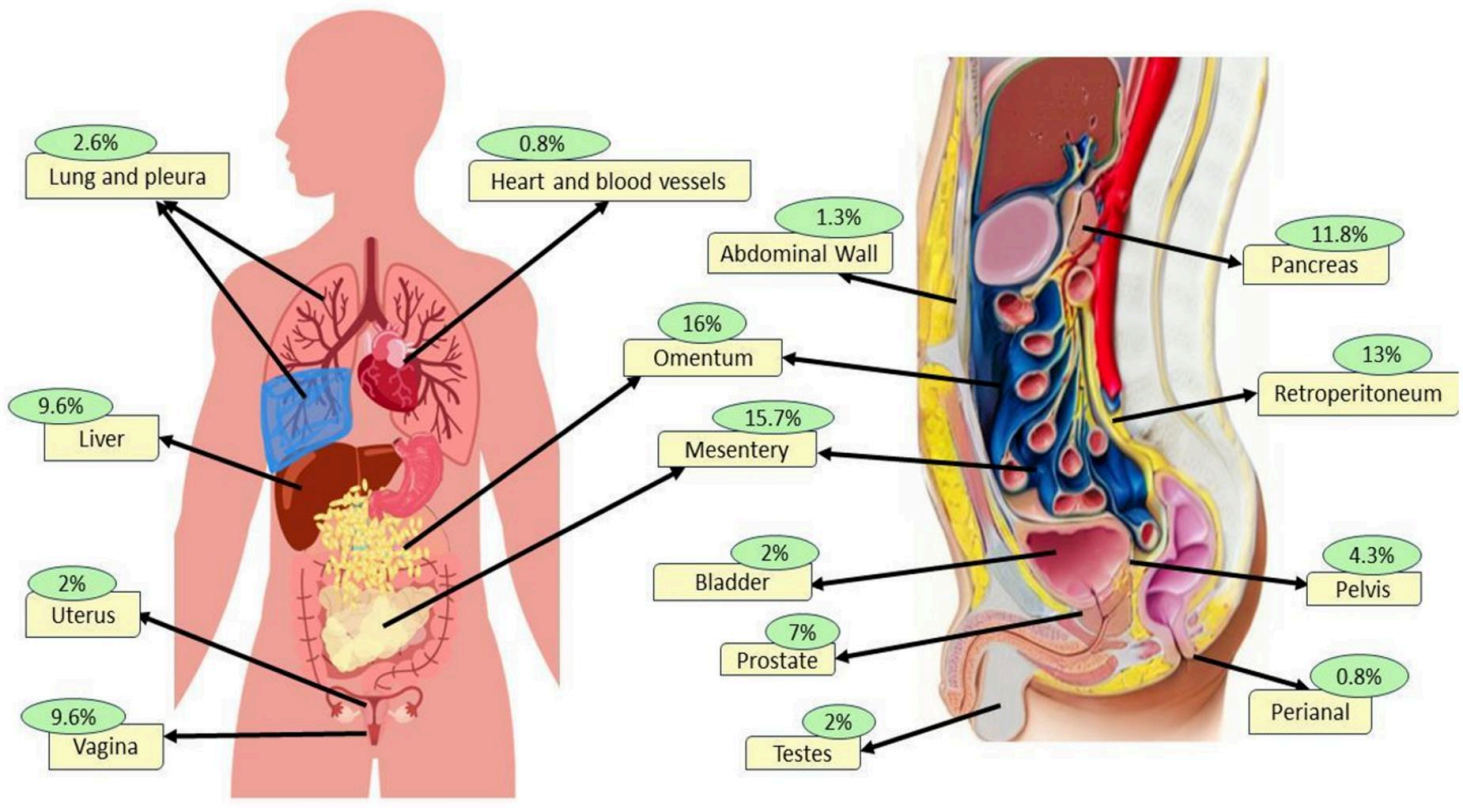
Frequencies of extragastrointestinal stromal tumors in different organs.

After a detailed and systematic review of the literature, 230 cases of EGISTs were identified. Standardized data were collected for tumor site, gender, age at presentation, presenting complaint, largest tumor dimension, number of tumors, mitotic rate, Ki-67 index, and histological and immunohistochemical patterns for each case (Table 3) [52–238]. The most frequently reported locations for these tumors were the omentum (16%), mesentery (16%), retroperitoneum (13%), pancreas (12%), liver (9.6%), vagina (9.6%), and prostate (7%) (Figure 2).

**Table 3.** Detailed summary of EGIST tumor characteristics, including size, location, histopathology, and immunohistochemistry.

Location	**Omentum** [52, 83–112]	**Mesentery** [109, 112, 113–134]	**Retroperitoneum** [53, 28, 54–57, 109, 111, 135–147]	**Pancreas** [58–63, 111, 148–167]	**Hepatic** [27, 64, 65, 167–184]	**Vaginal** [66–80, 185–188]	**Prostate** [81, 82, 189–202]
**Number of cases in literature**	37	36	30	28	22	22	16
**Male (%)**	58.3	52.8	44.8	50	57.1	0	100
**Female (%)**	41.6	47.2	55.2	50	42.9	100	0
**Age at presentation, mean + SD (years)**	55.5 ± 17.3	51.6 ± 15.6	60.1 ± 13.3	55.9 ± 11.8	57.6 ± 15.5	54.9 ± 16.3	54.5 ± 13.3
**Common presenting complaints**	Abdominal pain, 64% Abdominal mass, 27% Asymptomatic, 15% Abdominal distention, 12%	Abdominal pain, 61% Abdominal mass, 52% Abdominal distention, 30% Weight loss, 30% Anorexia, 22%	Abdominal pain, 61% Abdominal mass, 43% Asymptomatic, 13% Abdominal distention, 13%	Abdominal pain, 50% asymptomatic, 31% Weight loss, 27% Anorexia, 19% Anemia, 19% Fatigue, 15% Abdominal mass, 12% Back pain, 12%	Asymptomatic, 43% Abdominal pain, 43% Anorexia, 19%	Vaginal mass, 33% Vaginal bleeding, 28%	Urinary urgency, frequency, hesitancy, 63% Dysuria, 44%
**Largest tumor dimension, mean + SD (cm)**	14.3 ± 7.7	14 ± 5.3	15.8 ± 6.5	10 ± 7	13.3 ± 5.1	7 ± 3.9	10.3 ± 3.5
**Unifocal (%)**	73.9	70	80	73.9	70	90.4	N/A
**Multifocal (%)**	26	25	20	8.7	30	9.5	N/A
**Multicentric (%)**	0	5	0	17.3	0	0	N/A
**Spindle/fusiform**	61.7	71.4	75	76	65	100	85.7
**Epithelioid/polygonal/ovoid**	26.4	11.4	8.3	8	10	0	14.2
**Mixed**	11.7	14.3	16.6	16	25	0	0
**Myxoid**	0	2.8	0	0	0	0	0
**Median mitotic rate (per 50 HPF)**	5 (1–7.7)	5 (3–12.5)	5 (5–8.7)	5 (3–5)	10 (5–22.7)	10 (4–15)	5 (3–5.75)
**Median Ki-67 proliferation index**	5 (3–10)	8 (5–13.75)	6.5 (2.75–10)	40 (21.5–50)	6 (2–11.2)	8 (3.8–12.5)	5 (1–6.25)
**Prevalent immunohistochemical pattern**	CD117, 89% CD34, 61% SMA, 32% Vimentin, 25% DOG1, 21% Desmin, 11%	CD117, 100% CD34, 32% DOG1, 16% SMA, 16% Vimentin, 13%	CD117, 67% CD34, 52% S100, 38% Desmin, 33% SMA, 29% Vimentin, 24% DOG1, 14%	CD117, 92% CD34, 60% DOG1, 24% Vimentin, 20% SMA, 16% S100, 12%	CD117, 91% CD34, 55% DOG1, 32% SMA, 27% Vimentin, 23%	CD117, 100% CD34, 71% DOG1, 52% Vimentin, 29% Caldesmon, 24%	CD117, 100% CD34, 100% DOG1, 60% SMA, 33% Vimentin, 27%

Uterus [203–207], lungs and pleura [208–212], abdominal wall [213–215], bladder [216–219], testes [220–224], cardiovascular [225, 226], pelvic cavity [109, 227–236], perianal [237, 238].

### Omentum

A total of 37 cases of omental EGISTs were identified. Most patients with omental tumors presented with abdominal pain, distension, and mass, while some were asymptomatic. The male-to-female ratio for these tumors was 1.4:1. The mean age at presentation was 55.5 ± 17.3 years and the largest reported tumor dimension was 14.3 ± 7.7 cm. The majority (73.9%) of these tumors were unifocal and ∼26% were multifocal. Based on histological features, 61.7% had spindle cell morphology, 26.4% had epithelioid cell morphology, and 11.7% demonstrated mixed spindle and epithelioid cell patterns. The median mitotic rate was calculated to be 5/50 HPFs (interquartile range [IQR]: 1–7.7) and the median Ki-67 proliferation index was 5 (IQR: 3–10). CD117 and CD34 were positive in 89% and 61% of tumors, respectively.

Omental EGISTs can remain clinically silent for a long time despite their large tumor size because of their anatomic site and extramural growth. Small tumors are often incidentally detected during imaging studies for unrelated clinical indications. According to the National Institutes of Health algorithm for assessing malignancy in classical GISTs, most omental EGISTs would be classified as high-risk due to their large size alone. However, tumor size is not deemed a reliable prognostic parameter in the case of omental EGISTs [51].

EGISTs of the omentum usually present as large, well-circumscribed, post-contrast heterogeneously enhanced tumors with areas of necrosis and hemorrhage [52]. It is difficult to differentiate an EGIST in the omentum from a GIST of the lesser curvature of the stomach, even with advanced radiological imaging techniques. About half of omental EGISTs are misdiagnosed as extramucosal tumors of the stomach [239].

In a Chinese cohort of 27 omental EGISTs, 33% harbored the KIT mutation and 51.9% the PDGFRA mutations [25]. This is in contrast to the typical GISTs, in which PDGFRA mutations are identified in only 8%–10% of cases [29]. In a study on omental EGISTs, the positivity rates of CD117, CD34, and SMA were 93%, 61%, and 32% in solitary omental GISTs, and were 95%, 33%, and 14% in multifocal omental GISTs, respectively. In the survival analysis of the study, the median survival for multifocal omental GISTs was only 8–9 months without chemotherapy [8].

There were seven reported cases in which omental EGISTs presented with intra-abdominal tumor rupture and bleeding. In all cases, the tumors originated from the greater omentum and the diameter exceeded 10 cm in six cases. These observations suggest that large EGISTs originating from the greater omentum may increase the risk of tumor-associated bleeding [240].

### Mesentery

The most common presenting complaints among the 36 cases of mesenteric EGISTs were abdominal pain, distention, mass, weight loss, and anorexia. The male-to-female ratio was nearly 1:1 and the mean age at presentation was 51.6 ± 15.6 years. The largest tumor dimension was 14 ± 5.3 cm. Among these tumors, 70% were unifocal, 25% were multifocal, and 5% were multicentric. The frequencies of the histological patterns were 71.4% spindle, 11.4% epithelioid, 14.3% mixed, and 2.8% myxoid. The median mitotic rate was 5/50 HPFs (IQR: 3–12.5) and the median Ki-67 was calculated to be 8 (IQR: 5–13.7). CD117 was positive in 100% of these tumors, while only 32% were positive for CD34.

Mesenteric EGISTs tend to have a poor prognosis and a more aggressive course compared with omental EGISTs [8, 241]. Fletcher’s criteria classified mesenteric EGISTs as low-risk in 17% of cases, intermediate-risk in 5%, and high-risk in 78%. Tumor recurrence occurred in 21% of patients. Consistently with the omental EGISTs, Rammohan *et al.* [242] identified KIT mutations in only 27.8% of mesenteric EGISTs, with a higher frequency of PDGFRA mutations (38.9%). Follow-up data were available for 31 patients; 8 patients had tumor recurrence and/or metastasis. The mean tumor dimension, age, and histological subtypes were similar between the disease-free and recurrence groups. The mean mitotic rate was 8.5/50 HPFs in the disease-free group and 15.5/50 HPFs in the recurrence group.

### Retroperitoneum

Thirty cases of retroperitoneal EGISTs were reported. The male-to-female ratio was 1:1.2 and the mean age at presentation was 60.1 ± 13.3 years. Common presenting complaints were abdominal pain, mass, and distention, while 13% of patients were asymptomatic at diagnosis. The tumors were quite large, with the mean largest tumor dimension being 15.8 ± 6.5 cm. Of these, 80% were unifocal and 20% were multifocal. Spindle cell histology accounted for 75% of the cases, followed by epithelioid (8.3%) and mixed-type (16.6%). The median mitotic rate was 5/50 HPFs (IQR: 5–8.7), and the median Ki-67 proliferation index was 6.5 (IQR: 2.75–10). CD117 and CD34 were positive in 67% and 52% of the cases, respectively.

Retroperitoneal EGISTs are known to have poorer OS and disease-free survival than EGISTs occurring at other locations [243, 244]. Miettinen *et al.* [245] reported a median survival of 14 months, with only a few patients surviving beyond 5 years. The use of imatinib or other tyrosine kinase inhibitors [245] has significantly improved survival, increasing median survival from 18 to 60 months [246]. Zhou *et al.* [40] evaluated 22 cases of EGISTs originating from the omentum, mesentery, and retroperitoneum. Stratified survival analysis showed that EGISTs originating from the retroperitoneum had the worst prognosis, while those arising from the omentum had the most favorable prognosis [40].

Retroperitoneal EGISTs measuring <15 cm have better disease-free survival and OS compared with larger tumors when adjusted for survival outcomes and mitotic count of >5/50 HPFs, respectively [243]. Tumor size, however, does not help in determining the retroperitoneal EGIST invasiveness.

Lin *et al.* [28] found a correlation between exon 10 mutations of the KIT gene and tumor invasiveness with poor prognosis, whereas exon 11 mutations were associated with better disease-free prognosis [28] attributed to a more favorable response to imatinib [247].

Surgery is the gold standard of care for EGISTs and GISTs [248]. Barreda Bolanos *et al.* reported that surgical resectability is the most important prognostic factor [249]. Retroperitoneal EGIST invasion of the colon [54], mesentery, bladder, and ureter [55] can have disease-free outcomes if effectively resected and treated. However, if vascular invasion is present and surgery is not feasible, then prognosis is generally poor [56]. Khobragade *et al.* [57] reported successful resection of a retroperitoneal tumor involving the renal vein by performing a nephrectomy concurrently.

A high mitotic index or high Ki-67 labeling index is associated with poor prognosis [245–247]. Delayed diagnosis of retroperitoneal EGISTs leads not only to larger sizes of tumors, but also to more aggressive histological forms, characterized by increased cellularity, nuclear atypia, and pleomorphism [243].

### Pancreas

A total of 27 cases of pancreatic EGISTs were identified. The presenting symptoms included abdominal pain, weight loss, anorexia, anemia, fatigue, abdominal mass, and back or flank pain. Notably, 31% of patients were diagnosed incidentally and had no symptoms at presentation. The male-to-female ratio was 1:1 and the mean age at presentation was 55.9 ± 11.8 years. The largest tumor size was 10 ± 7 cm. Among these tumors, 73.9% were unifocal, 8.7% were multifocal, and 17.3% were multicentric. As observed in other EGIST locations, spindle cell histology was the most prevalent, with 76% of tumors exhibiting it, while epithelioid and mixed cell types accounted for 8% and 16% of cases, respectively. The median mitotic index was 5/50 HPFs (IQR: 3–5) and the median Ki-67 was 40 (IQR: 21.5–50). CD117 and CD34 positivity were seen in 92% and 60% of tumors, respectively.

A review of 19 cases of pancreatic EGISTs showed that 8 of 19 (42.1%) tumors occurred in the head of the pancreas, 5 in the tail (26.3%), 4 were involved both the body and tail (21.1%), 1 (5.3%) occurred in the uncinate process, and 1 (5.3%) involved the entire pancreas [58]. Abdominal CT, ultrasound, and endoscopic ultrasound (EUS) can help in determining the tumor localization, size, invasion of surrounding organs, and distant metastases. Most of them, however, are non-diagnostic [59]. EUS is the most successful at differentiating the tumor origin between the duodenum and pancreas, as it can accurately distinguish the different layers of the gastrointestinal tract. Preoperative EUS-fine-needle aspiration (EUS-FNA) or ultrasound-guided FNA (US-FNA) are efficacious additions to the diagnostic and treatment algorithm for pancreatic tumors [250]. The diagnostic value of tumor markers such as CA19-9 and carcinoembryonic antigen (CEA) for pancreatic EGISTs is limited and rarely used [60].

Surgical resection is the primary preferred treatment for pancreatic EGISTs [251], with the goal of achieving complete resection with clear safety margins [60]. Selecting the optimal type of surgical resection depends on the pancreatic EGIST location. Pancreatecoduodenectomy is the optimal treatment for pancreatic head tumors, while distal pancreatectomy is preferred for pancreatic tail tumors [60]. Routine lymph node dissection is not indicated in pancreatic EGIST cases because of rare regional lymph node metastases [61].

Beltrame *et al.* [62] described the follow-up information for 19 cases of pancreatic EGISTs. Disease recurrence following surgery was reported in five cases: three patients who underwent re-resection were alive and tumor-free after 30, 41, and 48 months. Overall, 18 patients were alive with a median survival time of 17.5 months (range, 1–66 months). Despite the limited number of cases and the short follow-up period, it appears that the resection of pancreatic EGISTs may offer a good prognosis, even in recurrent disease [62]. By univariate analysis, none of the features (including sex, age, tumor size, and cell type), except mitoses, was a significant predictor for recurrence and metastasis in pancreatic EGISTs [63].

### Liver

A total of 22 patients with EGISTs in the liver were reported. The most common presenting complaint was abdominal pain. Surprisingly, about half of the patients had no symptoms at diagnosis despite the large tumor size. The male-to-female ratio was 1.3:1 and the mean age at diagnosis was 57.6 ± 15.5 years. The largest tumor dimension was 13.3 ± 5.1 cm. The majority (70%) of the tumors were unifocal and 30% were multifocal. In terms of the histological patterns, 65% of tumors had spindle cell morphology, 10% had epithelioid cell morphology, and 25% had features of both subtypes. The median mitotic index was quite high for these tumors, at 10/50 (IQR: 5–22.7) HPFs, and the median Ki-67 index was 6 (IQR: 2–11.2). CD117 was found positive in 91% of tumors, CD34 in 55%, DOG1 in 32%, SMA in 27%, and vimentin in 23%.

Hepatic EGISTs are very difficult to diagnose clinically and radiographically. The majority of the cases are asymptomatic and are only diagnosed incidentally, either as an abdominal mass on routine physical examination or at later stages when the mass exerts space-occupying effects. On imaging, EGISTs often display heterogenous, cystic, or solid components, making them difficult to distinguish from other hepatic tumors [27]. The most accurate approach for diagnosing hepatic EGISTs is by using a combination of histological, immunohistochemical, and molecular studies on tissue samples. Positive staining for CD117 and CD34 can aid in confirming the diagnosis of these EGISTs [27].

Complete surgical resection of the tumor with negative margins is the optimal treatment option. For tumors that are potentially unresectable or have a large tumor burden, a customized approach including a combination of transarterial embolization, cytoreductive surgery, and adjuvant or neoadjuvant systemic chemotherapy should be considered [27, 64]. According to a study on primary hepatic EGISTs, 76.7% of the patients survived for >6 months after local hepatectomy alone or in combination with chemotherapy [252].

Tyrosine kinase inhibitors (TKIs) such as imatinib and sunitinib are promising adjuvant and neoadjuvant therapies for EGISTs. Long-term survival of 135 months was reported for a patient with a hepatic EGIST [65]. This patient was initially treated with argon-helium laser cytoreductive surgery for tumor burden reduction, followed by imatinib continuously for 61 months. Subsequently, he was treated with transcatheter embolization and sunitinib for 71 months to prevent disease progression [65]. In general, 88% of patients developed tumor recurrence ∼2 years after imatinib treatment [253]. The median survival time for patients with completely resected hepatic EGISTs was longer than that of those with incomplete resection or inoperable tumors [253].

### Vagina

A total of 22 cases of vaginal EGISTs have been reported in the literature. Presenting complaints of patients included vaginal mass and bleeding. The mean age at diagnosis was 54.9 ± 16.3 years and the largest tumor dimension measured 7 ± 3.9 cm. Among these tumors, 90.4% were unifocal and 9.6% were multifocal. All 22 cases (100%) demonstrated the spindle type of histology. All tumors reported positivity for CD117, 71% of cases were positive for CD34, 52% for DOG1, 29% for vimentin, and 24% for caldesmon. The median mitotic count was 10/50 (IQR: 4–15) HPFs and the median Ki-67 was 8 (IQR: 3.8–12.5).

It is difficult to differentiate the origin of vaginal EGISTs from the rectum or from the rectovaginal septum. Among the reported cases of 25 rectovaginal tumors, 6 originated from the rectovaginal septum [66–68, 254, 255], 8 from the vaginal wall [66–71, 255, 256], 2 from the vulvar region [82, 83], and 3 from the rectum [84–87]. In the remaining cases, the site of origin could not be confirmed either due to a lack of surgical or pathological data or adherence to both the rectal and vaginal walls [71, 254, 257, 258]. GISTs arising from the rectovaginal septum and vaginal wall are classified as EGISTs while those with rectal origin are considered GISTs. Regardless of the origin, these tumors were found to have worse prognoses than GISTs arising from the GI tract [90]. When a suspicious rectovaginal soft-tissue mass is identified, it is recommended to obtain a transvaginal biopsy for histo-immunochemical analysis and then proceed with complete surgical resection followed by adjuvant chemotherapy [259].

### Prostate

Prostatic EGISTs were relatively rare, with only 16 reported cases noted. Mean age at presentation for these tumors was 54.5 ± 13.3 years and the largest tumor dimension was 10.3 ± 3.5 cm. The most common presenting complaints were lower urinary tract symptoms and dysuria. Among the tumors, 85.7% had spindle cell histology and 14.2% were epithelioid. The median mitotic rate for these tumors was 5/50 (3–5.75) HPFs and the median Ki-67 was 5 (1–6.25). CD117 and CD34 were positive in all tumors, while DOG1, SMA, and vimentin were positive in 60%, 33%, and 27% of cases, respectively.

Most patients with primary EGISTs of the prostate had lower urinary tract symptoms such as dysuria, hematuria, urinary urgency, and retention. Occasionally, it presented as pain in the perineal region and a grossly enlarged prostate gland [81]. In a review of 15 patients with prostatic EGISTs by Li *et al.* [81], 93% of the patients had prostate-specific antigen (PSA) values within the normal range. This finding supported the theory that EGISTs of the prostate arise from the Cajal mesenchymal cells. The PSA level remains normal in EGISTs because PSA is mainly secreted by the human prostatic epithelial cells.

Immunohistochemistry plays a critical role in the diagnosis of prostatic EGISTs. DOG1 is strongly expressed on the cell surface of prostate EGISTs but is less frequently expressed in other soft-tissue tumors. Surgical resection remains the primary treatment for non-metastatic prostatic EGISTs. Tumor size and mitotic activity are significant prognostic factors. Imatinib mesylate is used as adjuvant therapy for advanced, unresectable, and metastatic cases. Patients with c-kit mutations in exons 8 and 11 were found to have a better response to imatinib therapy. Conservative treatment with imatinib alone appears to be beneficial for patients who decline or are unable to undergo surgery [81]. There are limited data to accurately predict the malignant potential of prostatic EGIST. Post-operative surveillance with abdominopelvic CT scan is important to identify early recurrent lesions [82].

## Conclusions

GISTs are the most common mesenchymal tumors of the gastrointestinal tract, typically arising in the stomach and small intestine, and are characterized by activating mutations in KIT or PDGFRA, which play a central role in tumorigenesis and therapeutic response.

EGISTs carry distinct clinical, pathological, and prognostic characteristics compared with GISTs. While both share key molecular features, including KIT and PDGFRA mutations, EGISTs generally present later, are more frequently misclassified, and carry poorer survival outcomes. Traditional prognostic markers, such as tumor size, are less reliable in certain EGIST sites, with mitotic index, nuclear atypia, and necrosis playing more central prognostic roles. Surgical resection remains the mainstay of treatment for both GISTs and EGISTs, with adjuvant TKIs, particularly imatinib, improving outcomes in high-risk or unresectable cases. However, EGISTs pose unique clinical challenges, including diagnostic delays due to extramural growth and the need for individualized treatment strategies that go beyond standard GIST models.

Future research should focus on developing site-specific prognostic models, improving molecular characterization of rare EGIST subtypes and expanding targeted therapeutic options beyond existing TKIs to address the unique biological and clinical challenges posed by these rare tumors.

## Authors' contributions

D.T.-Y.L., F.U., and R.R. conceived of the study and participated in its design and coordination. F.U., R.R., K.H., M.R., K.U.R., and I.W.K. collected clinical data and performed the pathological review. F.U., R.R., K.H., K.U.R., and I.W.K. carried out the statistical analysis and interpretation. D.T.-Y.L., F.U., R.R., K.H., M.R., K.U.R., and I.W.K. drafted the manuscript. All authors critically revised the manuscript, and all authors read and approved the final version of the manuscript.
